# A COVID-19 self-isolation monitoring module for FMUI undergraduate medical students: Linking learning and service needs during the pandemic surge in Indonesia

**DOI:** 10.1371/journal.pone.0279742

**Published:** 2022-12-30

**Authors:** Retno Asti Werdhani, Ardi Findyartini, Dewi Anggraeni Kusumoningrum, Chaina Hanum, Dina Muktiarti, Oktavinda Safitry, Wismandari Wisnu, Dewi Sumaryani Soemarko, Reynardi Larope Sutanto

**Affiliations:** 1 Department of Community Medicine, Faculty of Medicine Universitas Indonesia, Jakarta, Indonesia; 2 Medical Education Center, Indonesian Medical Education & Research Institute (IMERI) Faculty of Medicine Universitas Indonesia, Jakarta, Indonesia; 3 Department of Medical Education, Faculty of Medicine Universitas Indonesia, Jakarta, Indonesia; 4 Department of Child Health, Faculty of Medicine Universitas Indonesia – Dr. Cipto Mangunkusumo Hospital, Jakarta, Indonesia; 5 Department of Forensic and Medicolegal, Faculty of Medicine Universitas Indonesia – Dr. Cipto Mangunkusumo Hospital, Jakarta, Indonesia; 6 Department of Internal Medicine, Faculty of Medicine Universitas Indonesia – Dr. Cipto Mangunkusumo Hospital, Jakarta, Indonesia; 7 Undergraduate Program in Medicine, Faculty of Medicine Universitas Indonesia, Jakarta, Indonesia; Pondicherry Institute of Medical Sciences, INDIA

## Abstract

To ensure that students continued receiving adequate yet safe clinical exposure during the COVID-19 pandemic, the Faculty of Medicine at Universitas Indonesia (FMUI) created the Module of COVID-19 Self-Isolation Monitoring which aims to equip students with the knowledge and skill to monitor confirmed and close contact cases of COVID-19. Module development, divided into four phases: preparation, orientation, implementation, and evaluation phase, started as soon as the delta wave of COVID-19 cases forced medical students to halt their offline clinical rotations. A quantitative secondary data were obtained from student and patient satisfaction questionnaires and on students’ performance and reflection. We analyzed the distribution of module evaluation, the student’s discussion score during the module, the students’ interest in participating as Covid-19 volunteers before and after the module’s deployment, and the correlation between learning outcomes and satisfaction. A total of 372 patients were monitored by 208 students during the 4-week module. The response rates were above 80%, with the majority agreeing that students found this module well-organized and fulfilled their expectations. There was a significant increase in discussion scores from weeks 1 to 4, a significant difference in the proportion of students interested in COVID-19 volunteering before and after the module completion as well as a significant low correlation between the patient’s monitoring score sheet and the reflection essay towards the patient’s satisfaction. We should still improve tutors’ time management, tutors’ provision of triggering questions for critical thinking skills, and tutors’ feedback for students. The module met patient expectations and is expected to assist tutors in providing feedback and examples of doctor–patient communication, thus accelerating students’ competence in patient interaction. Further evaluation is needed regarding knowledge transfer, the impact on community health, and the faculty development program, especially regarding how tutors fulfill their roles as medical educators.

## Introduction

As of 7 July 2021, a total of 324,597 active cases of COVID-19 had been detected in Indonesia, with new cases reaching 31,000 per day. The disease severity varies, ranging from asymptomatic to critical, across all age groups. The number of deaths from COVID-19 has increased as the active cases numbers keep peaking. This disease has been responsible for 61,868 deaths, with an average of 500 deaths per day [1]. The high number of COVID-19 cases has caused shortages of facilities, including wards to manage COVID-19 patients. As of 28 June 2021, the bed occupancy ratio in DKI Jakarta (the province with the most COVID-19 cases in Indonesia) reached 90.69%, while the national isolation and ICU occupancy ratio was 49.99% [2]. Amid shortages of COVID-19 wards, self-isolation monitoring programs in patients’ residences have become one promising solution for asymptomatic and mild cases. During self-isolation, primary healthcare providers monitor symptom improvement and provide access to medicines through telemedicine and home visits, thus aiming to reduce COVID-19 deaths [3]. The COVID-19 pandemic also caused limitations in the learning process for medical students, especially in the clinical phase. In many countries, medical schools have had to withdraw their students from clinical rotation to ensure their safety [4, 5], limiting their exposure to clinical case management and direct interactions with patients [6].

Adaptations of teaching and learning activities for medical students during the pandemic, including those in the clinical settings, have been widely reported [7]. Concerns have been raised about students’ clinical skills attainment, competence to manage clinical cases and interact with patients empathetically, and professional development during this time [8, 9]. Several adaptations of learning methods in clinical clerkship have been implemented to support medical education during the pandemic. One of these adaptations is telemedicine, which refers to the use of communication technology to provide healthcare services at a distance [10]. In medical education, telemedicine can engage medical students in patient care, especially in times of crisis [11]. The use of telemedicine in monitoring home quarantine and isolation for COVID-19 patients has been proven to reduce the risks of delayed hospitalization due to disease progression [12]. Telemedicine can be incorporated into the curriculum to assist students in developing core competencies in patient care and communication, as well as medical knowledge, to further improve their decision-making and involve them in system-based practices [4, 13].

Due to the many active COVID-19 cases in Indonesia from June to August 2021, clinical clerkship was halted temporarily. To ensure that students continued receiving adequate yet safe clinical exposure, the Faculty of Medicine Universitas Indonesia created a module, titled *Modul Pemantauan Isolasi Mandiri Pasien* Coronavirus Disease (COVID-19)/Module of COVID-19 Self-Isolation Monitoring. This module aims to overcome the shortage of patient exposure among clinical-rotation students during the COVID-19 pandemic, especially during the devastating delta-COVID-19 virus outbreak. This module was designed to allow all clinical rotation students to communicate with and monitor patient during self-isolation. Through this module, students are expected to practice communication skills, identify problems, provide information, and refer (if symptoms worsen) without requiring face-to-face meetings under the supervision of the teaching staff. It is believed that students can still apply their clinical reasoning and critical thinking abilities through long-distance communication.

This module aims to equip students with the knowledge and skills to monitor patients who need self-isolation for confirmed COVID-19 cases and quarantine for close contact. Considering the program workflow and safety, medical students were involved in online monitoring through telemedicine under tutor supervision and expert guidance. Given that clinical students already have the skill sets to contribute according to their competency, this module also provided a good chance to nurture their professional identity as future doctors through participation in monitoring self-isolation program volunteers.

Despite the need for medical schools to adapt and provide meaningful learning experiences for future medical doctors, limited empirical studies have highlighted this concern during the pandemic. The incorporation of telemedicine in undergraduate medical curricula initiated by students or faculty has been described in different reports [13–19] with notes on the need of stronger evidence on the effectiveness [20, 21], as reported studies within the last three years haven’t delineated the results of program evaluation. Therefore, this study aimed to fill this gap by providing empirical evidence on the effectiveness of the clinical practice module, which was part of a fast response during the COVID-19 pandemic involving collaboration between teachers and students. This study will describe a clinical practice module highlighting students’ clinical experience in monitoring COVID-19 patients in home isolation through a telemedicine platform. The study also aimed to evaluate students’ satisfaction with the module and the tutors’ facilitation, students’ performance in the group, students’ participation in developing COVID-19 public health education media, students’ monitoring completion and self-reflection essays, and patient’s satisfaction with students’ monitoring. We used the Kirkpatrick framework (levels 1 and 2) [22] to evaluate the module comprehensively.

## Method

This study was approved by the Research Ethics Committee of the Faculty of Medicine Universitas Indonesia (KET-1215/UN.2F1/ETIK/PPM.00.02/2021). Participants provided written consent through e-form which explained that all the data obtained through the module will be further analysed by maintaining data confidentiality.

### Context

As technology develops, telemedicine has been introduced as a new form of clinical practice. Telemedicine offers many benefits by eliminating distance barriers and expanding health services to those with limited access [23]. However, this practice has not been integrated into undergraduate medical education curricula, including the Faculty of Medicine at Universitas Indonesia (FMUI). Since 2012, FMUI has implemented a 5.5-year curriculum, consisting of a 3.5-year preclinical stage and a 2-year clinical stage. During their clinical years, students are exposed to practice in academic hospitals; the new module is the first to expose students to telemedicine for clinical practice systematically. Aside from its main aim to equip students with the knowledge and skills to monitor COVID-19 patients in self-isolation, this module was also expected to introduce students to telemedicine-based practice, such as teleconsultation, professionalism in telemedicine, and managing ethical dilemmas that might occur.

### Description of the module

This module was conducted over 4 weeks, from July–August 2021. Students participating in this module were fourth-year medical students of the Undergraduate Medical Program of FMUI. The total of 208 students were divided into 29 groups, with 7–8 in each. They have passed clinical rotations in basic medicine and surgery and taken their clerkship oath. The rising COVID-19 wave forced these students to halt their offline rotations. This completely online module aims to give them the necessary clinical skills in a safe, distance-learning method with learning activities such as: introductory lectures, small group discussion with tutor, plenary with resource person, and structured assignment activities (self-isolation patient monitoring, self-reflection, develop educational material), seen in Table 1. These learning activities were facilitated through google sheet form^®^, educational material videos, covid-19 guideline documents, reflection sheets, and formative tests.

**Table 1:** Summary of module methods of self-isolation monitoring for coronavirus disease 19 (COVID-19) Faculty of Medicine Universitas Indonesia (FMUI).

No	Activities	Method	Platform	Information
1.	Introductory Lecture	Synchronous	Zoom^®^	Held on the first day of the running module
2.	Small group discussion with tutor	Synchronous	Zoom^®^	Held at least 1 time per week for Synchronous discussions via Zoom, and Asynchronous discussion through group conversations
3.	Plenary with the resource person	Synchronous	Zoom^®^	Held once per week
4.	Structured assignment activities	Asynchronous	EMAS^®^, GDrive ^®^, Google Sheet ^®^	Consist of 2 individual assignments and 1 group assignment
5.	Independent activities	Asynchronous	EMAS ^®^, GDrive ^®^	Reading assignments of various materials regarding Covid-19

**Fig 1:**
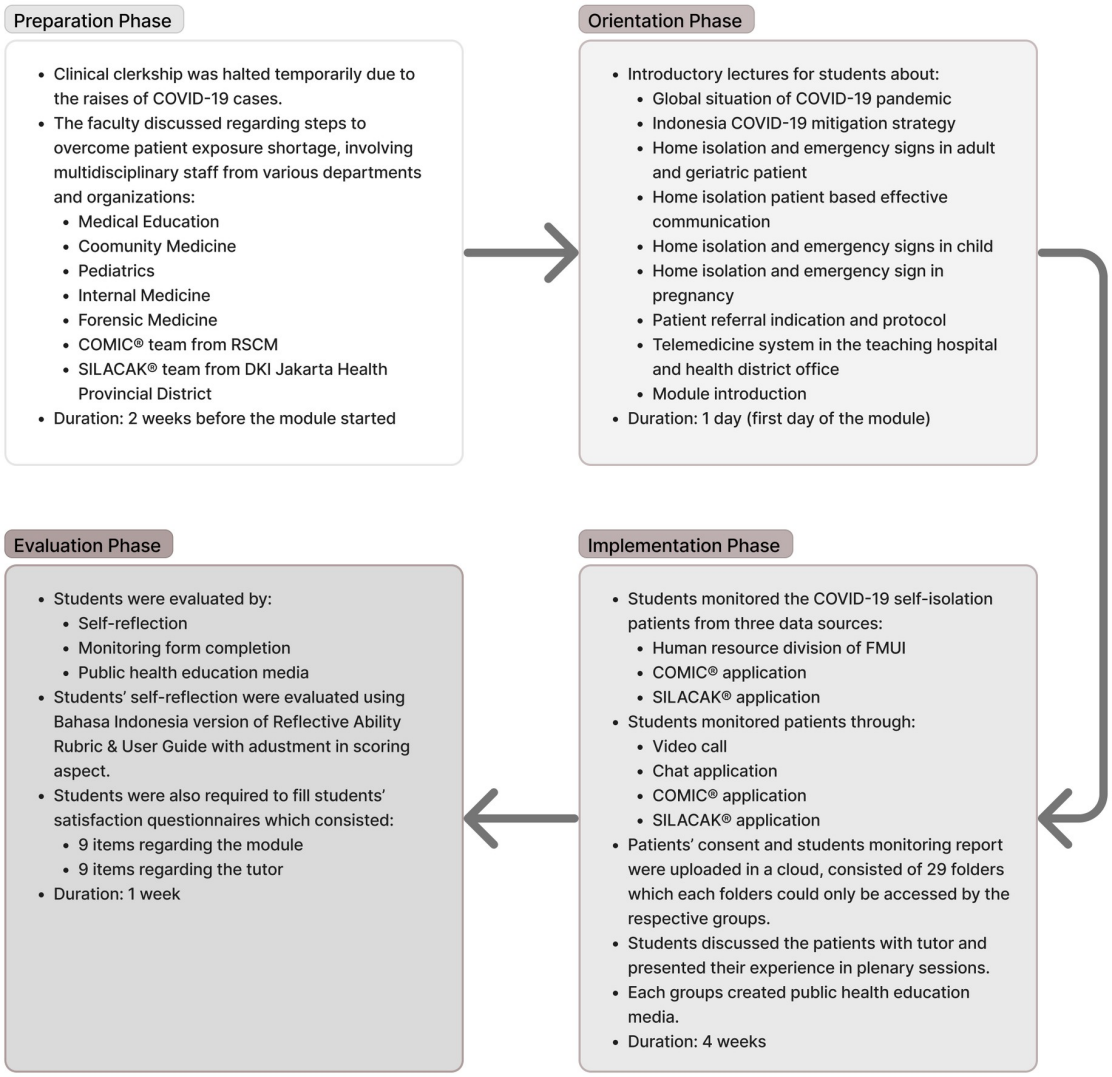
The summary of module organization phases.

The modules were organized in four phases: preparation phase, orientation phase, implementation phase, and evaluation phase. The summary of each phase illustrated in Fig 1.

#### Preparation phase

The academic calendar allocates four weeks for this module and is mandatory for all students in clinical rotation. Two weeks were allocated to the preparation of modules before students implemented them. The module team comprises multidisciplinary staff from different departments: medical education, community medicine, child health, internal medicine, and forensic medicine. With a ratio of 1 tutor to 7–8 students, 29 groups of students were formed, and each group has one tutor who comes from different departments. In addition, we also discussed the module design and students’ involvement in patient monitoring through telemedicine with the organizer of the COMIC^®^ system from *Cipto Mangunkusumo* Hospital and SILACAK^®^ system by the Ministry of Health, Republic of Indonesia organized by the DKI Jakarta health provincial district.

#### Orientation phase

The students were given a series of introductory lectures synchronously through the Zoom^®^ platform. Essential topics such as classification, pathophysiology, clinical symptoms (including emergency conditions), progression of Covid-19 and its current management in adults, children, and elderly, monitoring for COVID-19 patients, fulfilling the criteria for home isolation, self-isolation procedures and requirements, referral protocol for Covid-19, and telemedicine system from the teaching hospital and health district office, were covered during orientation phase. These lectures were conducted on the first day of the module, covering the natural history of COVID-19 and its management. Lecture materials and references regarding those topics were uploaded to Google Drive^®^ and E-learning Management Systems (EMAS)^®^ Universitas Indonesia, a learning management system developed by the university. Students were also required to complete a pretest in EMAS. This helped evaluate their understanding of COVID-19 and self-isolation before monitoring patients’ self-isolation.

#### Implementation phase

**Fig 2:**
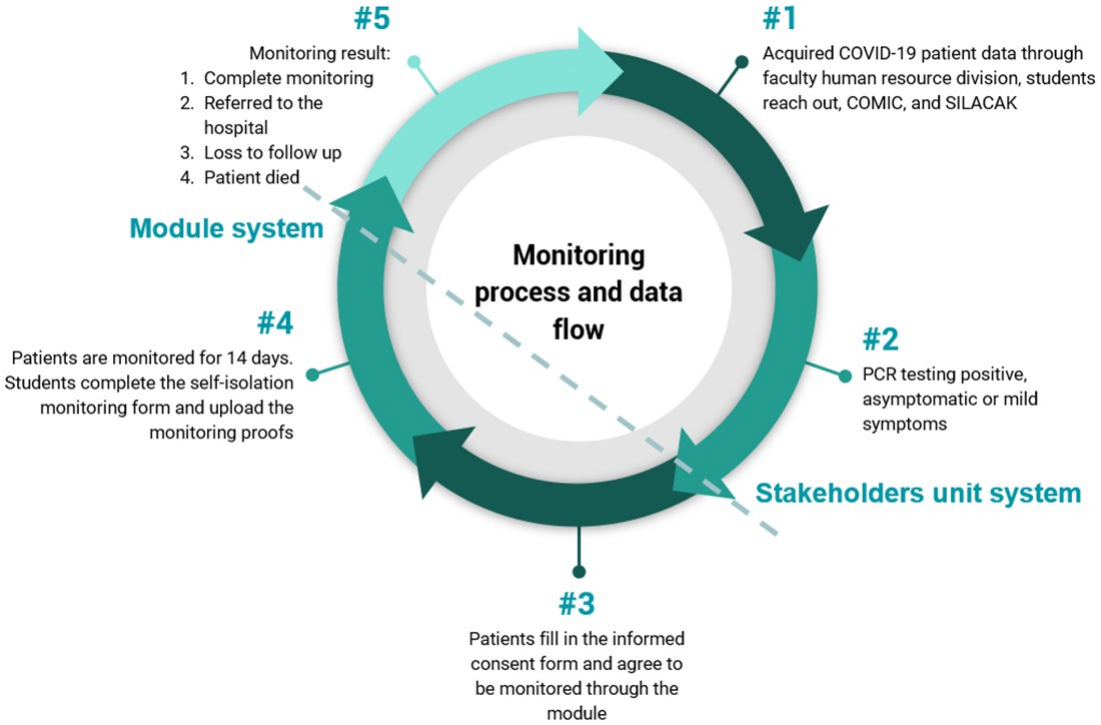
Monitoring process and data flow of the module.

This module was organized to enrich students’ experience in interacting with patients through safe clinical exposure while clinical clerkship was halted temporarily due to the pandemic. Students were required to monitor a minimum of one COVID-19 self-isolation patient who was asymptomatic or showed mild symptoms. These patients were identified from three data sources: the human resource division of FMUI and student contact, the COVID-19 Monitoring and Information Center (COMIC^®^) application, and the *Sistem Informasi Pelacakan* (SILACAK^®^) application. Telemonitoring was performed through telemedicine, i.e., video call, chat application, COMIC^®^ (developed by Dr. Cipto, Mangunkusumo Hospital, a teaching hospital affiliated with FMUI), and SILACAK^®^ (a COVID-19 tracing information system developed by the Ministry of Health, Republic of Indonesia). The monitoring process and flow of the module are provided in Fig 2.

Students were required to obtain each patient’s consent before monitoring. The patient data could only be accessed by the respective groups to ensure confidentiality. Before monitoring, students downloaded the self-isolation monitoring sheet provided in EMAS^®^. They were required to complete the sheet daily until the patients fulfilled the discharge criteria. The module team provided 29 folders that could only be accessed by the respective groups. Students were allowed to upload the daily self-isolation monitoring sheet and monitoring proofs, i.e., chat screenshots, audio recordings, or video call recordings. A total of 29 academic staff members were involved as tutors for the groups. Each tutor was also granted access to the students’ folder to evaluate the students’ progress in monitoring. Students then discussed their follow-up patients, as well as recommendations, knowledge input, and feedback once a week synchronously with their tutors through zoom or other platforms. The students presented their weekly reflections on the monitoring progress, support, and challenges, along with key questions, in 2 hour plenary sessions conducted every Friday with resource person from departments of internal medicine, child health, pulmonology, anesthesiology and emergency medicine, forensic, and community medicine as well as resource person from the COMIC^®^ and SILACAK^®^ systems. They were also required to create educational media in groups. Topics included universal precautions, health promotion, vaccination, and adverse events following immunization. Educational media could include public posters, educational videos, social media posts, podcasts, or articles in mass media.

#### Evaluation phase

The learning objectives in this module include (1) Explaining the requirements and procedures for self-isolation, (2) Explaining the pathophysiology of clinical symptoms and the course of Covid-19 disease, (3) Monitoring patient symptoms through telemedicine and effective communication, (4) Reviewing signs and symptoms of the disease, dangers, and procedures for referring patients to Covid-19 referral health facilities, and (5) Providing education about Covid-19, especially on aspects of prevention for patients and families. The achievement of learning objectives was measured using standard evaluation instruments from the faculty whose content has been reviewed by the academic staff, as seen in Table 2 below:

**Table 2:** Standard evaluation instruments for student’s evaluation.

No	Learning Objectives	Instrument
1.	(1), (2), (3), (4)	Patient monitoring assessment form (score 1–100) Group discussion assessment form (score 1–100)
2.	(3), (5)	Self-reflection assessment form (score 1–100)
3.	(5)	Educational media assessment form (score 1–100)

Evaluation of the implementation of the module was carried out using several validated and reliable instruments. The validation of the evaluation instrument for module implementation was carried out with Exploratory Factor Analysis (EFA) on all students participating in the module (208 students) with the results of the validation and reliability are presented in Table 3. They were considered valid and reliable considering the results of the EFA analysis (KMO value > 0.8 and p < 0.05, variance explained (AVE) > 0.5, with factor loading values > 0.4 and Cronbach alpha/Internal consistency > 0.8 [24].

**Table 3:** Module implementation evaluation instrument.

No	Module evaluation form	Number of items	Indicator measured	Validation and Reliability
1.	Student’s satisfaction towards module	9 items (self-developed)	Agree or disagree	Cronbach alpha 0.924
				Factor loading 0.609–0.870 (AVE 0.623, KMO sampling adequacy 0.935, p < 0.001
2.	Student’s satisfaction towards tutor	9 items (self-developed)	Agree or disagree	Cronbach alpha 0.885
				Factor loading 0.637–0.846 (AVE 0.560, KMO sampling adequacy 0.865, p < 0.001
3.	Patient’s satisfaction towards students	8 items (adapted from Fadhilah et al. [25]	Agree or disagree	Cronbach alpha 0.977
				Factor loading 0.886–0.956 (AVE 0.861, KMO sampling adequacy 0.904, p <0.001

### Design and data collection

This observational study aimed to evaluate the module using quantitative secondary data that were obtained from students’ satisfaction questionnaires and patients’ satisfaction questionnaires. This study also evaluated students’ increase in knowledge through small group discussion scores, self-reflection scores, and educational media scores, and patient monitoring completion scores scored by tutors. At the end of the module, students were asked about their experience of becoming volunteers during the Covid-19 Pandemic before and after the module. All data obtained were analyzed using IBM SPSS version 20.

### Data analysis

We analyzed the score distribution of module evaluation as well as learning objective scores during the module. Descriptive data were presented as percentages on categorical variables and mean on numeric variables/scores. The Friedman test was conducted to determine the significant increase in discussion scores from weeks 1 to 4. The Mc-Nemar test was completed to determine the significant difference in the proportion of students interested in COVID-19 volunteering before and after the module completion. The correlation test was conducted to determine the correlation between the patient’s monitoring score sheet and the reflection essay toward the patient’s satisfaction.

## Results

### Patients

**Fig 3:**
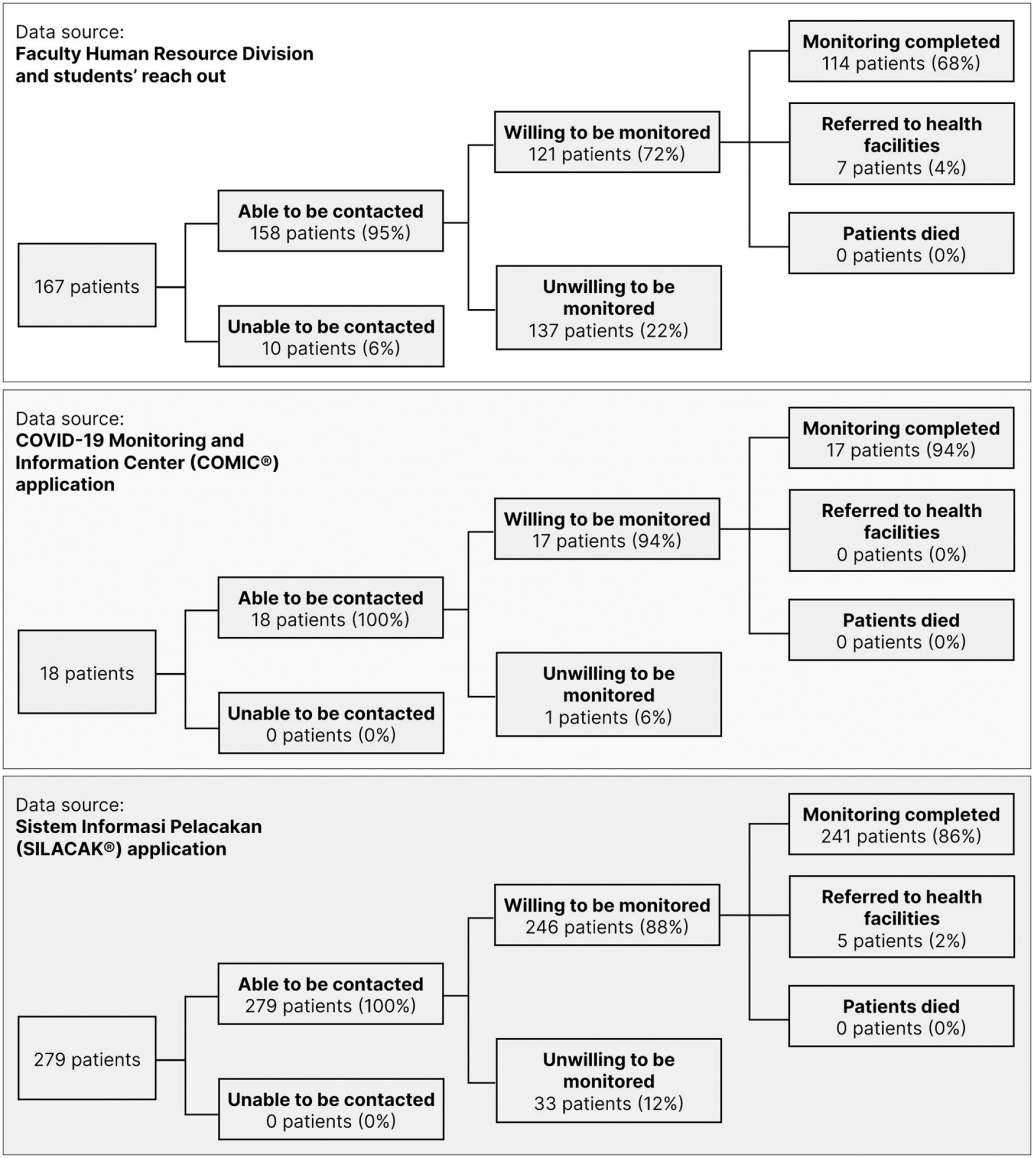
Patient monitoring cohorts of the 4-week module.

A total of 372 patients were monitored by 208 students during the 4-week module. The patient cohorts are illustrated in Fig 3.

### Students

A total of 208 students were enrolled in this module: 84 (40.38%) men and 124 (59.62%) women with age range between 21–23 years old. They were in their first year of clinical rotation and had taken the clerkship oath.

#### Students’ satisfaction with module implementation

Table 4 presents the students’ satisfaction with the module implementation. The response rate of the questionnaire was 87%, with most answers being positive (mostly agree), indicating that students found this module was well-organized and fulfilled their expectations and needs to achieve the competencies. Coordination of patient mapping with the module team, COMIC^®^ team, and SILACAK^®^ team had the highest proportion of positive answers (87%). This showed the importance of teamwork and bridging communication between the module team and students as well as with other stakeholders (*Cipto Mangunkusumo* Hospital for the COMIC^®^ team and the Provincial Health Office for SILACAK^®^). However, we also noted that the participation of teaching staff as tutors and resource persons could be greatly improved in the future, along with the need for more student involvement and support in the module management; shown by the lowest proportion of positive answers, only 62% students agree that the teaching staff (tutors, introductory lecture resource persons, plenary resource persons, field tutors) carried out their duties well.

**Table 4:** Students’ satisfaction toward module implementation (N = 208).

No	Items	Disagree	Agree
1	Competencies stated in the student guidebook were achieved upon completion of this module.	58 (27.9%)	150 (72.1%)
2	The learning resources and media in this module were adequate.	51 (24.5%)	157 (75.5%)
3	The teaching staff (tutors, introductory lecture resource persons, plenary resource persons, field tutors) carried out their duties well.	79 (38%)	129 (62%)
4	The module team prepared the module well.	58 (27.9%)	150 (72.1%)
5	The module team provided enough chance to enrich the self-isolation monitoring experience.	66 (31.7%)	142 (68.3%)
6	Module secretariats supported the learning process.	64 (30.8%)	144 (69.2%)
7	Student involvement in module management supported the implementation of the module.	78 (37.5%)	130 (62.5%)
8	The self-isolation monitoring sheet was easy to use.	49 (23.6%)	159 (76.4%)
9	Coordination of patient mapping with the module team, COMIC team, and SILACAK team went well.	27 (13%)	181 (87%)

#### Students’ satisfaction with tutors

Table 5 describes the students’ satisfaction with tutors. The questionnaire response rate was 100%. Overall, the students agreed that the tutors were helpful during the module. However, we should improve tutor time management, asking and responding to questions to trigger critical thinking, and giving feedback to students.

**Table 5:** Students’ satisfaction with tutors (N = 208).

No	Items	Disagree	Agree
1	Tutor allocates enough time to discuss with students	2 (1%)	206 (99%)
2	Tutor responds to students’ questions adequately	0	208 (100%)
3	Tutor is easily contacted	0	208 (100%)
4	Tutor responds to students’ questions regarding self-isolation/self-quarantine important aspects	2 (1%)	206 (99%)
5	Tutor is friendly and open	0	208 (100%)
6	Tutor facilitates the self-isolation/self-quarantine monitoring well	0	208 (100%)
7	Tutor gives appropriate moral support to students during monitoring	0	208 (100%)
8	Tutor gives constructive feedback	2 (1%)	206 (99%)
9	Tutor asks questions that trigger students to think critically	6 (2.9%)	201 (97.1%)

#### Students’ reflection on supporting and inhibiting factors in monitoring patients

Regarding self-isolation monitoring, the students were asked to reflect on the supporting and inhibiting factors they experienced through a weekly reflection form. Table 6 presents the data from the weekly reflections up to 4^th^ week. The weekly reflection response rates were 87.98% up to week 4. Overall, the students agreed with the supporting factors and disagreed with the inhibiting factors. Regarding inhibiting factors, a sudden increase occurred in students’ disagreement that “instructions from the module team are not clear” in week 3. The results show that the module was conducted according to students’ expectations for support and without meaningful obstacles and was improved along its implementation. In the plenary sessions, students also shared that the dynamic telemedicine environments with challenges in navigating communications with COVID-19 patients with different conditions in their home isolation can be overwhelming and may introduce ethical dilemmas to students; such as when the family asks the students of worsening patients to find a referral to hospital, even though it was not their responsibility or patients who refuse for referral due to his/her worsen the condition, even though the students have tried their best.

**Table 6:** Supporting and inhibiting factors in monitoring patients up to week 4 (N = 208).

Item	Disagree	Agree
Supporting factors		
Patients are enthusiastic	53 (25.5%)	155 (74.4%)
Tutors support the monitoring process	28 (13.5%)	180 (86.5%)
Module team gives clear instructions	30 (14.4%)	178 (85.6%)
Resource persons give clear explanations	26 (12.5%)	182 (87.5%)
Inhibiting factors		
Patients are unable to communicate	174 (83.7%)	34 (16.3%)
Tutors are difficult to contact	206 (99%)	2 (1%)
Instructions from the module team are not clear	197 (94.7%)	11 (5.3%)
Explanations from resource persons are intricate	205 (98.6%)	3 (1.4%)
No monitoring device is available in patients’ residences	111 (53.4%)	97 (46.6%)
Patients are not cooperative	161 (77.4%)	47 (22.6%)

The majority of students agreed on all supporting factors, with the lowest positive answer percentage for the item “patients are enthusiastic” (74.4%). Conversely, most students disagreed on all inhibiting factors, with the lowest disagreed answer percentage for “no monitoring device is available in patients’ residences”. This might have been because most patients did not own monitoring devices, such as pulse oximeters and home blood pressure monitors.

#### Students’ learning outcome evaluation

The students were also evaluated through discussion sessions. Their mean scores from four times small group discussion with tutor were 81.953 (± 5.596), 82.518 (± 5.525), 82.878 (± 6.507), and 84.092 (± 6.368), with an overall mean of 82.860. We observed a significant increase in discussion scores from weeks 1 to 4 (p < 0.001). The minimum–maximum of all discussion scores was 52.5–97.5, with 99% of students’ scores above 65 (the pass limit value for undergraduate students). Students were also evaluated through the completion of the patient monitoring sheet (mean score 85.35 ± 9.990), reflection essay (mean score 87.95 ± 8.413), and educational media (mean score 84.08 ± 7.364). The final scores were calculated from discussion scores, self-reflection essay scores, completion of monitoring progress forms, and educational media assignment scores.

Students were asked whether they had experience as volunteers and whether they were interested to volunteer on completion of the module. Before the module began, there were a total of 106 students (51%) already had experience in COVID-19 volunteering, and 102 students (49%) did not. After the completion of the module, 144 students (69.2%) were interested in COVID-19 volunteering, and 64 (30.8%) were not. A significant difference existed in the proportion of students interested in COVID-19 volunteering before and after the module completion (51% vs. 69.2%, respectively, p < 0.001).

#### Patients’ satisfaction with student’s monitoring program

The proportions of patients’ answers are shown in Table 7.

**Table 7:** Patients’ satisfaction with student’s monitoring program (N = 208).

No	Items	Disagree	Agree
1	Listens well to patient’s complaints and symptoms	89 (42.8%)	119 (57.2%)
2	Shows concern to patient (does not show boredom or ignore content conveyed)	91 (43.8%)	117 (56.3%)
3	Answers patient’s questions well and gives patients chances to ask	89 (42.8%)	119 (57.2%)
4	Uses understandable language and avoids using medical terms when giving explanations	89 (42.8%)	119 (57.2%)
5	Asks patient’s willingness to be monitored through the program	87 (41.8%)	121 (58.9%)
6	Communicates things that must be done and obeyed by patient during self-isolation	81 (38.9%)	127 (61.1%)
7	Communicates signs of emergency that patients during self-isolation should be aware of	81 (38.9%)	127 (61.1%)
8	Gives continuous daily monitoring of patients during self-isolation	95 (45.7%)	113 (54.3%)

The questionnaire response rate was 61.1% (208 patients out of 372). Hence, patients felt satisfied with the monitoring service provided. However, the proportion of positive answers/agreed did not exceed 70% for any item. The range of positive answers was 54.3–61.1% for all items. This is an important note for the module team and faculty because this module is expected to assist tutors in providing feedback and examples in doctor–patient communication, thus accelerating students’ competence in interacting with patients.

There was a low correlation between the patient’s monitoring score sheet and the reflection essay towards the patient’s satisfaction. (r 0,246 with p < 0,001 and r 0,202 with p 0,003). Students with higher patient’s monitoring score sheets and reflection essays tended to have higher scores in patient’s satisfaction. This may be a phenomenon in which students who actively follow up and have a thorough grasp of the material tend to provide better education/monitoring, resulting in satisfied patients.

## Discussion

This study has described the development of a clinical practice module that assigned students to monitor COVID-19 patients in home isolation through a telemedicine platform provided by stakeholders (teaching hospital and provincial health district office). Overall, this study showed students’ satisfaction towards the module, the tutors’ support and facilitation as well as their involvement in the home isolation monitoring program. Students’ performance in the group discussions and in public health media production as well as in monitoring completion and students’ reflective essays demonstrated very satisfactory results, Patients being monitored by the students also showed overall good satisfaction towards students’ monitoring activities.

Given the need to sustain adequate clinical exposures for medical students during the pandemic and provide opportunities for medical students and the medical school to participate in COVID-19 pandemic control in DKI Jakarta actively, this module was expected to fulfil both expectations. This modules’ preparation, implementation and evaluation stages involved stakeholders, including medical students, medical teachers from different departments, and COVID-19 telemedicine teams from hospital and primary health care.

To our knowledge, this module is the first in Indonesia, and probably Asia, to involve students in monitoring COVID-19 patients while facilitating student–patient interaction and communication skills virtually. Reports on students’ run telemedicine programs during the pandemic for different purposes in patient care have been reported in different regions. For example, the medical student-run telemedicine clinic through an academic medical centre-operated syringe services program in Florida [26], the medical student-run telemedicine elective program to provide telehealth follow-up for recently discharged patients with COVID-19 in Texas [27], and teleconsultation activities under the supervision to improve clinical reasoning and evidence-based practices of medical students in Brazil [28]. The COVID-19 pandemic has caused limitations in medical students’ learning process, especially those in the clinical phase. In many countries, medical schools have had to withdraw their students from clinical rotation to ensure their safety [4, 5], thus limiting their exposure to clinical case management and direct interactions with patients [6]. Therefore, telemedicine-based modules implemented in this study might be a very good alternative to provide necessary clinical experience to students during the pandemic through hands-on and supervised telemonitoring experience [13, 29].

In this telemedicine-based module, students were expected to develop rapport through two-way communication in educating and reassuring patients. However, students might experience several limitations in communicating through telemedicine, such as internet access issues, lack of body language, difficulty expressing emotion, resistance to technology, or patient preference for face-to-face consultation [30], some of which also took place in the current study. Patients’ psychological state, such as feeling stress due to COVID-19 and being interviewed about it, might also explain why patients were less enthusiastic to be monitored through telemedicine [31]. This follows the result that the percentage of students who agreed with the statement of “patients are enthusiastic” were fewer than other statements as supporting factor. Additionally, students’ limited knowledge and experience causing poorly developed rapport might have contributed to the communication barriers. In addition to the patient’s medical condition, they are also more likely to be in a state of psychological stress (i.e., depression and anxiety) due to being infected by COVID-19 [26, 32].

Tutors must minimize communication limitations through intensive discussion and constructive feedback with students. Tutors’ experience and advice regarding doctor–patient communication help construct an ideal model of communication for students. However, their skills in giving constructive feedback and their ability to provoke students’ thoughts vary. Through students’ tutor satisfaction, we observed that some students felt their tutors did not give constructive feedback. This might be caused by the tutor not allocating enough time to discuss and give feedback to students [33], which was also reflected in the students’ answers for the item “tutor allocates enough time to discuss with students”. Students also might not feel they received constructive feedback because the feedback given by tutors tends to be one-way communication and was not necessarily in line with the students’ needs. Before giving constructive feedback, it is important to identify students’ skills and knowledge by asking questions and discussing their reflections on their experience [34]. However, the students’ responses revealed that some tutors did not ask questions that triggered them to think critically; thus, the tutor might fail to assess students’ clinical reasoning and critical thinking skills. This is an important note for the faculty to include this aspect in the faculty development program, especially regarding how tutors fulfill their roles as medical educators [35] and in optimizing provision of constructive feedback [33].

Through patient monitoring, students are expected to have learned about interprofessional collaboration. During the monitoring process, students were asked to identify internal factors (e.g. behavior, age, gender, self-hygiene, and nutrition) and external factors (e.g. family, work environment, and other environments) affecting patients’ health and wellbeing. They were also encouraged to map the various factors that caused patients to experience COVID-19 infection and advocate a recommendation plan to relevant stakeholders from different professions. This was intended to demonstrate that collaboration and good communication between various professions are needed to mitigate COVID-19 [36].

This study demonstrates how telemedicine-based practice could be integrated with current medical curricula and its impacts towards students’ doctor-patient communication skills in telemonitoring. Firstly, telemedicine allowed the students’ involvement in the COVID-19 mitigation plan as patient navigators and educators under tutor supervision while monitoring patients during their self-isolation period. As well as providing clinical practice using a new method, this module could reflect the medical student’s role during the pandemic [37]. Secondly, through this module, FMUI contributed to the government COVID-19 mitigation plan by monitoring patients’ self-isolation, thus breaking the transmission chain. Thirdly, this module demonstrates how online learning and telemedicine-based modules could help students to gain clinical experience when conventional clinical learning methods need to be temporarily halted due to the pandemic. Fourthly, this module became an example and success story in developing the curriculum, as the first that incorporated telemedicine-based practice into undergraduate clinical education. Conducting clinical practice through telemedicine has become an important competency to acquire as the “Medicine 4.0” era is a near future, which means technology will be the center pillar in diagnosing and managing patients [38]. Therefore, this approach should be integrated with current undergraduate clinical education systems, not only as a curriculum adaptation due to the pandemic but also to prepare students as future doctors facing Medicine 4.0. Lastly, monitoring systems (COMIC and SILACAK) are highly dependent on the amount and quality of data to operate well. This module also demonstrated the importance of big data in managing community health problems and designing comprehensive telemedicine systems [39]. In the future, students may take part in building Indonesia’s big data system that will be beneficial for healthcare services.

This study has practical and theoretical implications. First, a model of empowering students in helping to monitor self-isolating and self-quarantining COVID-19 patients can be a valuable asset in a national COVID-19 management strategy. Second, because the module was developed when COVID-19 cases were highest in Indonesia, it has added value and served as a prototype of how to involve students in a COVID-19 mitigation plan. Therefore, whenever needed, the module can be readily reinstated to allow students to contribute to the fight against COVID-19 while simultaneously performing their academic role as students. Third, this module highlights the importance of linking curriculum closer with the health system which was shown from the COVID-19 mitigation attempt involving different stakeholders. Finally, a careful plan and dynamic implementation are required to integrate telemedicine-based content in the curriculum. This includes consideration of relevant learning theories such as social cognitive and experiential learning theories in planning and implementing the telemedicine in the course. From the practice perspective, we also attempted to provide continuous support and debriefing sessions with the tutors and the resource person should the students face an ethical dilemma. At the same time, where possible, we also tried to communicate challenges encountered by the students through our stakeholders promptly.

This study has several limitations. It was based on one institution’s experience, and the module only involved one student batch, it might be difficult to generalize the result to other institutions. We did not have the baseline for students’ telemonitoring skills before the module hence pre-post test comparison was not possible. Moreover, this study evaluated the students’ satisfaction with the module and the tutors’ facilitation, and their performance in the group discussion, patient monitoring completion, public health education media and self-reflection essay, and patients’ satisfaction towards student monitoring. Even though such an approach was quite comprehensive, we realize that further evaluations regarding knowledge transfer in different telemedicine settings and the impact on community health levels.

## Conclusion

The Module of COVID-19 Self-Isolation Monitoring was developed as an immediate response to participate in COVID-19 mitigation and in providing clinical exposures to medical students in the clinical year despite limitations during the pandemic. The module has incorporated opportunities for the students, under supervision, to conduct telemonitoring of COVID-19 patients in home isolation. There was a significant increase in discussion scores from weeks 1 to 4 and a significant difference in the proportion of students interested in COVID-19 volunteering before and after the module completion.

Based on the comprehensive evaluation, the module has met students’ and patients’ expectations, with some important notes for future improvement including needs to increase capacity for tutors to provide feedback and examples in doctor–patient communication, thus accelerating students’ competence attainment in interacting with patients in telemedicine context. Further evaluations are needed to document knowledge transfer in different telemedicine settings and the impact on community health. Important implications of this study towards medical education in clinical years, especially in using telemedicine platforms, and in involving students to the real attempts of mitigating COVID-19 pandemic were also discussed.

## Supporting information

S1 Appendix Assessment form. (DOCX)

S2 Appendix Data availability. (XLSX)
