# Supplementary material for: A COVID-19 self-isolation monitoring module for FMUI undergraduate medical students: Linking learning and service needs during the pandemic surge in Indonesia
Source: PLoS One. 2022 Dec 30;17(12):e0279742. doi: 10.1371/journal.pone.0279742 (PMC9803291; doi:10.1371/journal.pone.0279742)
Supplement: S1 Appendix — (DOCX) [file pone.0279742.s001.docx]

**S1 Appendix.**

1. **Patient satisfaction assessment form**

Patient satisfaction assessment will be compiled in the form of a Google Form, which will be filled in by the patient after completing self-isolation.

Several criteria will be assessed in the Patient Satisfaction Assessment, including:

1. Communication (adapted from Fadhilah et al., 2011)

1. Greet in a friendly manner and always use polite language during carry out monitoring
2. Treating patients as equals and not demeaning patients
3. Able to listen to complaints and symptoms experienced by patients well
4. Shows concern with what is conveyed by the patient (does not show boredom or ignoring the patient's complaints)
5. Able to answer questions asked by patients well, and allow the patient to ask questions
6. Use easy-to-understand language, avoiding medical terminology while giving an explanation

2. Professionalism

a. Obtain informed consent early in the monitoring process

3. Self-Isolation Monitoring

- 1. Exploring daily patient data and other complaints that may be experienced

1. Telling things to do and obey during self-isolation
2. Communicating things that the patient must be aware of (emergency signs) during the self-isolate treatment
3. Monitoring continuously (every day) during the self-isolation period.

Reference :

Fadhilah M, Oda Y, Emura S, Yoshioka T, Koizumi S, Onishi H, et al. Patient satisfaction questionnaire for medical students’ performance in a hospital outpatient clinical: A cross-sectional study. Tohoku J Exp Med. 2011;225(4):249-54. doi: 10.1620/tjem.225.249.

**B. Group discussion assessment form**

**GROUP DISCUSSION ASSESSMENT FORM**

(FOR MODULE SECRETARIAT)

| Group : | Module : |
| --- | --- |
| Tutors’ name : | Academic Years : |
| Discussion : 1/2/3/4 |  |

| **No** | **Name** | **Participation** | | | | **Behavior** | | **Total (Max= 40)** |
| --- | --- | --- | --- | --- | --- | --- | --- | --- |
|  |  | Sharing | Argumentation | Active | Dominant | Discipline/ Absence | Communication |  |
| 1 |  |  |  |  |  |  |  |  |
| 2 |  |  |  |  |  |  |  |  |
| 3 |  |  |  |  |  |  |  |  |
| 4 |  |  |  |  |  |  |  |  |
| 5 |  |  |  |  |  |  |  |  |
| 6 |  |  |  |  |  |  |  |  |
| 7 |  |  |  |  |  |  |  |  |
| Etc.. |  |  |  |  |  |  |  |  |

Note:

| **Participation** | Score | | |
| --- | --- | --- | --- |
|  | **0-5** | **6-7** | **8-10** |
| Sharing | Fair | occasionally | Always |
| Argumentation | Fair | Enough | Good |
| Active | Fair | Enough | Good |
| Communication | Fair | Enough | Good |
| **Behavior** | Score | | |
|  | **-5** | **-3** | **0** |
| Dominant | Yes | occasionally | Tidak |
| Discipline/ Absence | Late >15’ | Late < 15’ | On Time |

**Definition of evaluation item:**

Sharing: sharing opinions/knowledge in accordance with the scope of discussion among group members

Argumentation: providing knowledge and logical responses based on the literature he reads

Activity: active in discussions without being encouraged by the facilitator

Dominant: the attitude of controlling the forum during group discussions

Communication: listening, explaining and asking questions using good and correct and systematic language

Jakarta, .......................................
Tutor,

( )

**C. Completeness assessment form for daily patient monitoring data**

**DAILY PATIENT MONITORING DATA COMPLETENESS ASSESSMENT FORM**

(FOR MODULE SECRETARIAT)

| Group : | Module : |
| --- | --- |
| Tutors’ name : | Academic Years : |
|  |  |

| **No** | **Name** | **Data Completeness** | | | **Total Score** |
| --- | --- | --- | --- | --- | --- |
|  |  | Monitoring data is completely filled in | Monitoring is carried out continuously until the criteria for completion of isolation / the patient dies / are referred to the health facility | Attach proof of daily documentation |  |
| 1 |  |  |  |  |  |
| 2 |  |  |  |  |  |
| 3 |  |  |  |  |  |
| 4 |  |  |  |  |  |
| 5 |  |  |  |  |  |
| 6 |  |  |  |  |  |
| 7 |  |  |  |  |  |
| 8 |  |  |  |  |  |
| 9 |  |  |  |  |  |
| 10 |  |  |  |  |  |
| 11 |  |  |  |  |  |

Note:

| **Data Completeness** | Score | | |
| --- | --- | --- | --- |
|  | **0-5** | **6-7** | **8-10** |
| Monitoring data is completely filled in | Fair | occasionally | Always |
| Monitoring is carried out continuously | Fair | Enough | Good |
| Attach proof of daily documentation | Fair | occasionally | Always |

#### Final Score = Total Score / 3

Jakarta, .......................................

Tutor,

( )

**D. Self-reflection assessment form**

**SELF-REFLECTION ASSESSMENT FORM**

(FOR MODULE SECRETARIAT)

| Group : | Module : |
| --- | --- |
| Tutors’ name : | Academic Years : |
| Self reflection : 1/1 |  |

Give the most appropriate score (between 0-100) for the self-reflection script prepared by the student and then provide WRITTEN FEEDBACK in the column below.

| **Score Range** | **Scoring Instruction** |
| --- | --- |
| 0 | There is a description of the learning experience but it does not match the trigger (prompt) |
| 55-59 | There is an appropriate description of the learning experience but no self-reflection |
| 60-64 | There are lessons learned that have been identified, but no significant relationship has been shown |
| 65-69 | Relying on self-assessment completely, without including external evidence that |
| 70-79 | Include external evidence supporting identified lessons learned |
| 80-89 | Explicitly refers to relevant previous experience and explains how previous experience affects the situation that occurs |
| 90-100 | Analysis includes external evidence supporting lessons learned, links to previous experiences, and implications for the future/next steps (action plan) |

Notes:

Self-reflection script assessment must still pay attention to aspects of the Indonesian language used in writing

**Feedback on student self-reflection scripts:**

Things that are correct:

Things that still need to be improved:

Jakarta, .......................................

Tutor,

( )

**E. Educational material assessment forms**

**EDUCATIONAL MATERIAL ASSESSMENT FORM**

(FOR MODULE SECRETARIAT)

| Group : | Module : |
| --- | --- |
| Assignment : 1/1 | Academic Years : |

| **No.** | **Name** | **Substance** | **Clarity** | **Display** | **Total  Score** |
| --- | --- | --- | --- | --- | --- |
| 1. |  |  |  |  |  |
| 2. |  |  |  |  |  |
| 3. |  |  |  |  |  |
| 4. |  |  |  |  |  |
| 5. |  |  |  |  |  |
| 6. |  |  |  |  |  |
| 7. |  |  |  |  |  |
| 8. |  |  |  |  |  |

If the student group does not submit assignments, Score = 0

**Definition of evaluation items:**

Substance: solid material (concise), clear, and comprehensive, in accordance with existing theory.

Clarity: the material is easy to understand, uses language or terminology that is easily understood by the public, and includes reference sources

Display: display of educational materials

Note :

|  | Score | | |
| --- | --- | --- | --- |
|  | **0-5** | **6-7** | **8-10** |
| Substance | Fair | occasionally | Always |
| Clarity | Fair | Enough | Good |
| Display | Fair | occasionally | Always |

#### Final Score = Total Score / 3

Jakarta, .......................................

Tutor,

( )
